# Lifetime Exposure to Estrogen and Neuroimaging outcomes in older women in the IGNITE trial

**DOI:** 10.1093/geroni/igaf122.2175

**Published:** 2025-12-31

**Authors:** Amber Watts, Robyn Honea, Shannon Donofry, Hayley Ripperger, Sarah Aghjayan, Chaeryon Kang, Kirk Erickson

**Affiliations:** University of Kansas, Lawrence, Kansas, United States; University of Kansas School of Medicine, Kansas City, Kansas, United States; RAND Corporation, Santa Monica, California, United States; University of Pittsburgh, Pittsburgh, Pennsylvania, United States; University of Pittsburgh, Pittsburgh, Pennsylvania, United States; University of Pittsburgh, Pittsburgh, Pennsylvania, United States; AdventHealth Research Institute, Orlando, Florida, United States

## Abstract

Lower lifetime estrogen exposure is associated with age- and Alzheimer’s-related reductions in brain volume, while estrogen-based therapies are associated with larger volume. We investigated associations between hormone-based medication use and structural neuroimaging markers, hypothesizing that hormone-based therapies would be associated with larger brain volumes. We investigated baseline associations in Investigating Gains in Neurocognition in an Intervention Trial of Exercise (IGNITE), a multi-site, randomized exercise trial. We report structural data from magnetic resonance imaging. We processed the T1-MPRAGE using SPM12 and CAT12 software, adjusting for total intracranial volume. We focused on regions related to Alzheimer’s disease and higher concentrations of estrogen-beta receptors, dominant in the hippocampus. In 459 women (ages 65-80), we evaluated associations between retrospective recall of hormone-based medication use (birth control, menopausal hormone therapy) and brain volumes accounting for age, education, SES, and APOE4 carrier status. Birth control use (average start age 22) was associated with larger right parahippocampal volume (β = 0.100, p = 0.034). APOE4 carriers had smaller volumes in estrogen-beta-receptor regions (β=-0.096, p = 0.045). APOE4 carriers using birth control had smaller volumes than non-carriers using birth control (β=-.242, p=.028). Younger age when starting menopausal hormone-therapy was associated with greater white matter volume (β=-0.150, p = 0.037). These cross-sectional findings support the critical window hypothesis, i.e., timing of estrogen exposure is important for healthy brain aging. Our study uniquely links estrogen exposure in early adulthood to later-life brain structure. Future research should investigate mechanisms by which APOE4 may moderate estrogen’s influence on brain aging. Findings have clinical implications for estrogen therapies throughout the lifespan.

